# Construction of competitive endogenous RNA network reveals regulatory role of long non‐coding RNAs in type 2 diabetes mellitus

**DOI:** 10.1111/jcmm.13224

**Published:** 2017-06-23

**Authors:** Zijing Lin, Xinyu Li, Xiaorong Zhan, Lijie Sun, Jie Gao, Yan Cao, Hui Qiu

**Affiliations:** ^1^ Department of Endocrinology and Metabolism the First Affiliated Hospital of Harbin Medical University Harbin Heilongjiang Province China; ^2^ Harbin Medical University Harbin Heilongjiang Province China

**Keywords:** type 2 diabetes mellitus, competitive endogenous RNA, long non‐coding RNA, mTOR, miR‐181b

## Abstract

Increasing epidemic of type 2 diabetes mellitus (T2DM) and its comorbidities makes it urgent to understand the pathogenesis and regulatory mechanism. However, little is known about the regulatory role of lncRNAs in diabetes. Here, we constructed a T2DM‐related competitive endogenous RNA (ceRNA) network (DMCN) to explore biological function of lncRNAs during the development of diabetes mellitus. This network contained 351 nodes including 98 mRNAs, 86 microRNAs and 167 lncRNAs. Functional analysis showed that the mRNAs in DMCN were annotated into some diabetes‐related pathways. Furthermore, mTOR‐centred subnetwork was extracted and ncRNA‐involved mTOR pathway was established. Finally, we validated that NEAT1 was potentially communicated with mTOR signalling target protein mLST8 *via* the association with miR‐181b. These findings provide significant insight into lncRNA regulatory network in T2DM.

## Introduction

The increasing epidemic of T2DM and its comorbidities urge our understanding of the pathogenesis and regulatory mechanisms of T2DM 1. The recent discovery of functional non‐coding RNAs (ncRNAs) as specific gene expression regulators suggests that manipulating ncRNAs could be a novel therapeutic approach for combating metabolic disorders such as diabetes mellitus 2. Numerous studies demonstrated that microRNAs have been implicated in diabetes mellitus 3. However, less is known about the regulatory role of lncRNAs in diabetes. Recent studies provide evidence that endogenous RNAs influence each other's levels by competing for a limited pool of microRNAs 4. ceRNA theory proposed that all types of RNA transcripts communicate through a new ‘language’ mediated by microRNA binding sites or microRNA response elements (MREs) 4.

Recently, various ceRNA networks have been constructed to gain a global view of regulatory interaction networks of ceRNAs in certain diseases. Yang *et al*. developed Starbase v 2.0 to systemically identify the RNA–RNA and protein–RNA interaction networks from 108 CLIP‐Seq data sets generated by tumour samples 5. The ceRNA theory has been applied to elucidate the pathogenesis of numerous diseases such as cancer, muscular dystrophy and neurodegenerative disease 6, 7, 8. However, little is known about potential role of ceRNAs in metabolic disease, especially diabetes mellitus.

In this study, we constructed a T2DM‐related ceRNA network (DMCN) to explore the biological functions of lncRNAs in the development of diabetes mellitus. After functional analysis, we found that the mRNAs in DMCN were annotated into some diabetes‐related pathways. Then, mTOR‐centred subnetwork was extracted and ncRNA‐involved mTOR pathway was established. Finally, we validated that NEAT1 was potentially communicated with mTOR signalling target protein mLST8 *via* the association with miR‐181b.

## Materials and methods

### Collection of human T2DM‐related genes

We downloaded human T2DM‐related genes from The Text‐mined Hypertension, Obesity and Diabetes candidate gene database (T‐HOD) 9 and Online Mendelian Inheritance in Man (OMIM)‘s Morbid Map 10. A total of 632 human T2DM‐related genes were obtained.

### Identification of microRNA and T2DM‐related gene pairs

The DIANA‐TarBase (V6.0) 11 and miRTarBase (V4.5) 12 databases collected the microRNA–mRNA interaction pairs, which were manually curated and experimentally verified. From these two databases, we downloaded human microRNA–mRNA pairs that were verified by qPCR and Western blot analysis. After integrating process, we obtained 43,497 non‐redundant microRNA–mRNA pairs. By integrating the collected human T2DM‐related genes, we then screened T2DM‐related microRNA–mRNA pairs from these non‐redundant microRNA–mRNA pairs.

### Identification of microRNA and T2DM‐related lncRNA pairs

The human microRNA–lncRNA interaction pairs were identified from starBase (V2.0) 5, which were supported by ClipSeq experiments. A total of 10,213 experimentally verified microRNA–lncRNA pairs were identified. By combining microRNAs with T2DM‐related target genes, we identified microRNA–lncRNA interaction pairs associated with T2DM.

### Construction of human T2DM‐related ceRNA network (DMCN)

The microRNA–lncRNA–gene interactions were integrated if microRNA–mRNA and microRNA–lncRNA pairs shared common microRNAs. By combining the collected T2DM‐related microRNA–mRNA pairs with T2DM‐related microRNA–lncRNA pairs, we identified 543 pairs of T2DM‐related microRNA–lncRNA–gene interactions and then constructed the human T2DM‐related ceRNA network.

### Topological features of T2DM‐related ceRNA network

The degree is the number of edges that connect to a certain node. *K* is the number of edges that connect to node *i*. It can be defined as follows: Deg (*i*) = *K*

For a graph G: = (V,E) with *n* nodes, the betweenness *B*_*i*_ stands for a node *i* as

$$B_{i}=\frac{1}{\left(n-1)(n-2\right)}\sum_{s\neq i\neq t}\frac{S_{st}\left(i\right)}{S_{st}}$$

where *S*_st_ is the number of shortest paths from s to t, and *S*_st_(*i*) represents the number of shortest paths from s to t that pass through a node *i*. The measure is normalized by the number of pairs of nodes except *i*, which is (*n*‐1)(*n*‐2).

### Function annotation of ncRNAs to mTOR signalling pathway

To investigate biological functions of T2DM‐related genes in the network, we used DAVID 6.7 for gene ontology (GO) functional annotation of T2DM genes and KEGG pathway for pathway enrichment 13. After GO enrichment by DAVID tool, the GO BP terms with FDR value <0.1 were chosen as statistically significant terms. KEGG (Kyoto Encyclopedia of Genes and Genomes, http://www.genome.jp/kegg/) is a collection of online databases. T2DM genes were subjected to KEGG pathway analysis, and FDR values <0.1 were considered as statistically significant pathways.

We manually draw figure of mTOR signalling according to KEGG pathway database and annotated mirRNAs and lncRNAs to this pathway according to their interactions with genes involved in mTOR signalling pathway. The mirRNAs and their target genes were downloaded from miRTarBase (http://mirtarbase.mbc.nctu.edu.tw), which is an experimentally validated microRNA–target interactions database 14.

### Cell culture

Human liver cell line HL7702 was purchased from the Type Culture Collection of the Chinese Academy of Sciences (Shanghai, China). Cells were grown in DMEM or RPMI‐1640 supplemented with 10% foetal bovine serum, 100 U/ml penicillin and 50 mg/ml streptomycin at 37°C with 5% CO_2_.

### Quantitative RT‐PCR

Total RNA was isolated, and complementary DNA (cDNA) was generated with the use of a High Capacity cDNA Reverse Transcription Kit (ABI, Applied Biosystems, Carlsbad, CA, USA). cDNA from miRNAs was then generated with the use of TaqMan miRNA Reverse Transcription Kit (GenePharma, Shanghai, China). Quantitative reverse transcriptase PCR (RT‐PCR) was conducted using TaqMan gene expression assays for NEAT1 MALAT1 and GAPDH or TaqMan Universal Master Mix II with TaqMan microRNA assay for miR‐181b, miR‐144‐3p and U6.

### Dual‐luciferase reporter assay

Two Luc‐NEAT1 constructs were generated including potential binding site (NEAT1‐WT) or mutant binding site (NEAT1‐Mut). HEK293 cells were seeded in 12‐well plates, cultured for 24 hrs and then cotransfected with NEAT1‐WT or NEAT1‐Mut plasmid and miR‐181b mimics or negative control sequences using Lipofectamine 2000 (Invitrogen, Carlsbad, CA, USA). The luciferase activity was measured 48 hrs after transfection using Dual‐Luciferase reporter assay system (Promega, Madison, WI, USA).

### Western blot analysis

Proteins from cells were separated by sodium dodecyl sulphate‐polyacrylamide gel electrophoresis (10–12%). After blocking, the membranes were probed with antibody for mLST8 (ab25974) and β‐actin (ab8227) (Abcam, Cambridge, UK) overnight at 4°C. The membranes were then incubated with infrared (IR) fluorescent dye‐labelled secondary antibody (Alexa Fluor; Molecular Probes, Eugene, OR, USA) for 1 hr. The bands were analysed using an IR Imaging System (LI‐COR Biosciences, Lincoln, NE, USA), and band density was quantified using Odyssey 3.0 software (LI‐COR Biotechnology, Lincoln, NE, USA) and normalized to β‐actin.

### RNA‐binding protein immunoprecipitation assay

RNA‐binding protein immunoprecipitation (RIP) assay was performed using EZ‐Magna RIP Kit (Millipore, Billerica, MA, USA) according to the manufacturer's instruction. The enrichment of miRNAs binding AGO2, a key component of the microRNA‐containing RISC complex, was used as the positive control, and U6 as the negative control. Briefly, cells were lysed with RIP lysis buffer, followed by incubated with RIP buffer containing magnetic beads conjugated with human anti‐Ago2 antibody (Millipore). Proteinase K was used to digest the protein, and then immunoprecipitated RNA was isolated. Purified RNA was subjected to qRT‐PCR analysis.

### Statistical analysis

All experiments were repeated three times. Data were shown as mean ± S.D. and analysed with a two‐tailed Student's *t*‐test. *P* ≤ 0.05 was considered statistically significant.

## Results

### T2DM‐related ceRNA network (DMCN)

**Figure 1:**
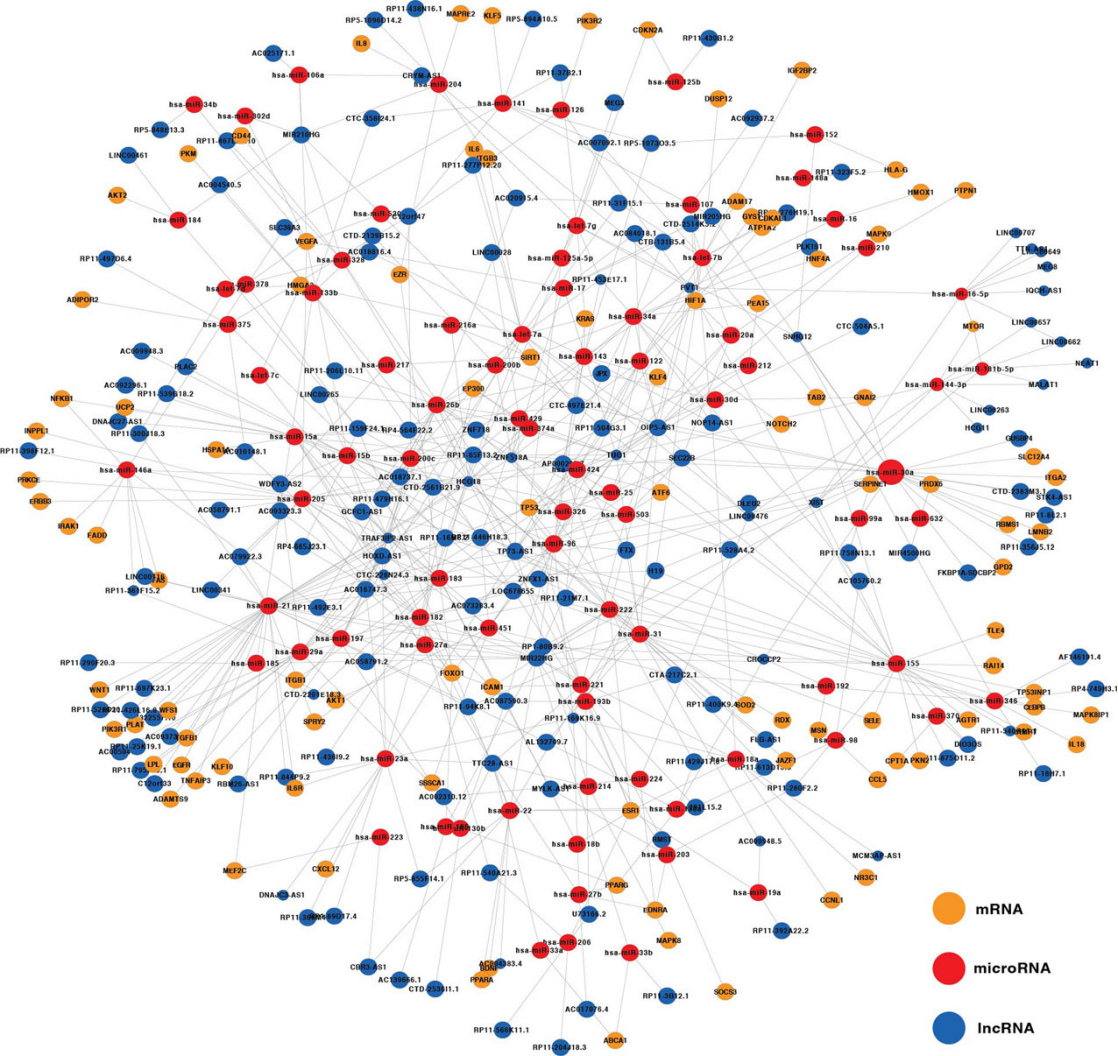
ceRNA network related to type 2 diabetes mellitus. Nodes in yellow, red and blue represented genes, microRNAs and lncRNAs, respectively.

**Figure 2:**
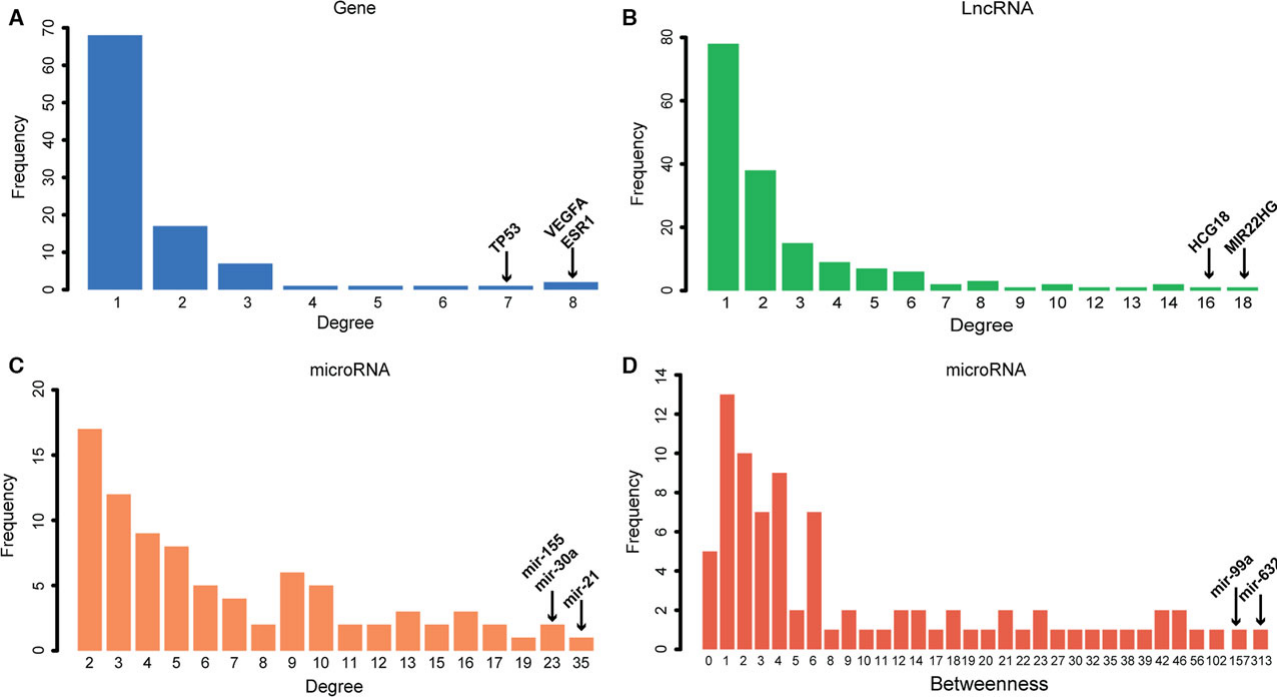
(**A**,** B** and **C**) showed the degree distribution of genes, lncRNAs and microRNAs, respectively. Horizontal axis represented the degree values, and vertical axis represented the number of nodes with a certain degree value. (**D**). Horizontal axis represented the betweenness value of microRNAs.

We constructed DMCN that included 98 genes, 86 microRNAs and 167 lncRNAs (Fig. S1). All 351 nodes formed a giant component (Fig. 1). A node's degree is defined as the total number of edges connecting it in the network (Fig. S2). Among the top 10 microRNAs with the highest degree, hsa‐mir‐21 was at top of the list (degree = 35), indicating 35 edges (35 targets) which directly connect to miR‐21. As for gene VEGFA (degree = 8) and lncRNA MIR22HG (degree = 18), eight edges and 18 edges are directly connected to VEGFA and MIR22HG, respectively. Node with the higher degree means that this node is highly connected and likely to be ‘hub’ node. Yellow, red and blue nodes represented type 2 diabetes‐related genes, microRNAs and lncRNAs, respectively (Fig. 1). The edges between the nodes represented the interactions between these RNAs. The size of each node represented the degree. The top 10 nodes of genes, microRNAs and lncRNAs with highest degree were shown in Table 1. The highest degree nodes were VEGFA/ESR1, hsa‐mir‐21 and lncRNA MIR22HG, respectively (Table 1 and Fig. 2A–C).

**Table 1:** The top 10 degree list of miRNAs, genes and lncRNAs

miRNA	degree	Gene	degree	LncRNA	degree
hsa‐miR‐21	35	VEGFA	8	MIR22HG	18
hsa‐miR‐155	23	ESR1	8	HCG18	16
hsa‐miR‐30a	23	TP53	7	OIP5‐AS1	14
hsa‐miR‐31	19	EP300	6	TUG1	14
hsa‐miR‐183	17	FOXO1	5	TRAF3IP2‐AS1	13
hsa‐miR‐26b	17	KRAS	4	TP73‐AS1	12
hsa‐miR‐15a	16	HMGA2	3	ZNF518A	10
hsa‐miR‐182	16	HIF1A	3	HOXD‐AS1	10
hsa‐miR‐29a	16	PPARG	3	TTC28‐AS1	9
hsa‐miR‐15b	15	EDNRA	3	RP11‐65F13.2	8

We also calculated a network feature‐node betweenness, defined as the fraction of shortest paths between node pairs that pass through a given node. The betweenness of a microRNA represents its role in connecting mRNAs and lncRNAs to each other (Fig. S2). In our DMCN, the higher betweenness value of a microRNA means that it regulates more potential ceRNA pairs. Our results showed that miR‐21, miR‐155, miR‐30a had not only higher degrees, but also higher betweenness values (Table 1), indicating their role not only in connecting other nodes, but also in the interactions with mRNAs and lncRNAs. However, miR‐632 and miR‐99a had the highest betweenness value (betweenness value = 1) but relative low degree (Table 1 and Fig. 2D), suggesting their role in connecting genes and lncRNAs rather than being a ‘hub’ node in a local network.

### Gene ontology and KEGG pathway analysis of T2DM genes

**Figure 3:**
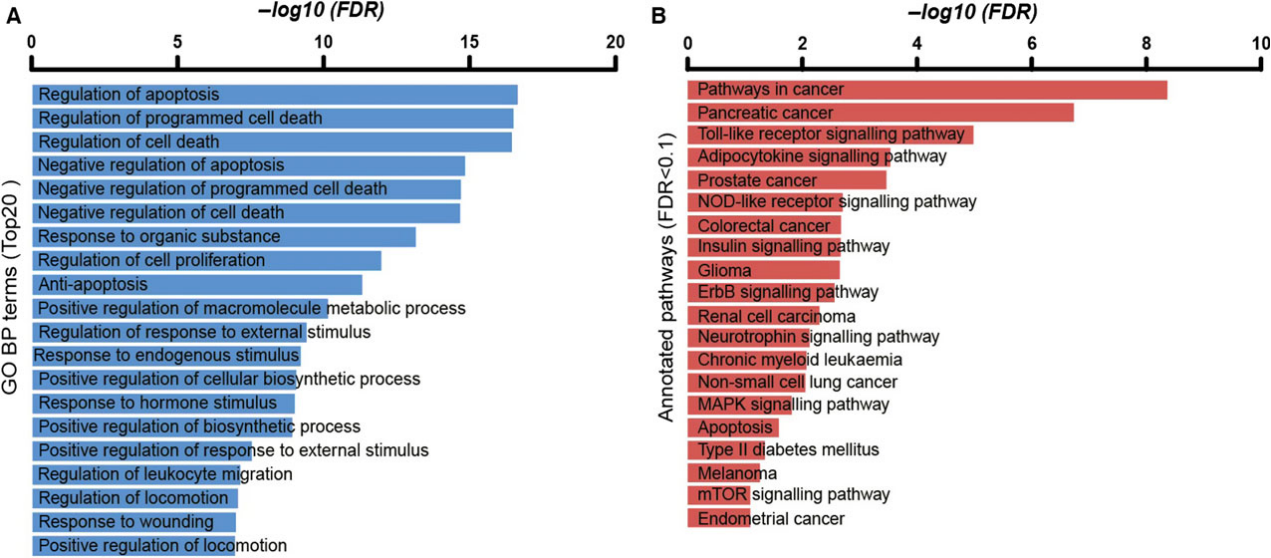
(**A**) Gene ontology (GO) analysis of T2DM‐related genes. (**B**) KEGG pathway analysis of T2DM‐related genes. Only top 20 terms or pathways were presented.

To explore biological functions of lncRNAs during the development of diabetes mellitus, we annotated DMCN into GO and KEGG pathways. The GO BP (biological process) analysis showed that the most significant enriched terms were bidirectional regulation of apoptosis and cell death, and others were related to response to external and internal stimulus and cellular metabolism (Fig. 3A). The top 20 most significant enriched pathways included pathways in cancer, insulin signalling pathway, type 2 diabetes and mTOR signalling (Fig. 3B). Among these 20 pathways, half were related to cancer, and others were mainly involved in immune response, energy and substance metabolism, neuronal function.

### The components of mTOR subnetwork

**Figure 4:**
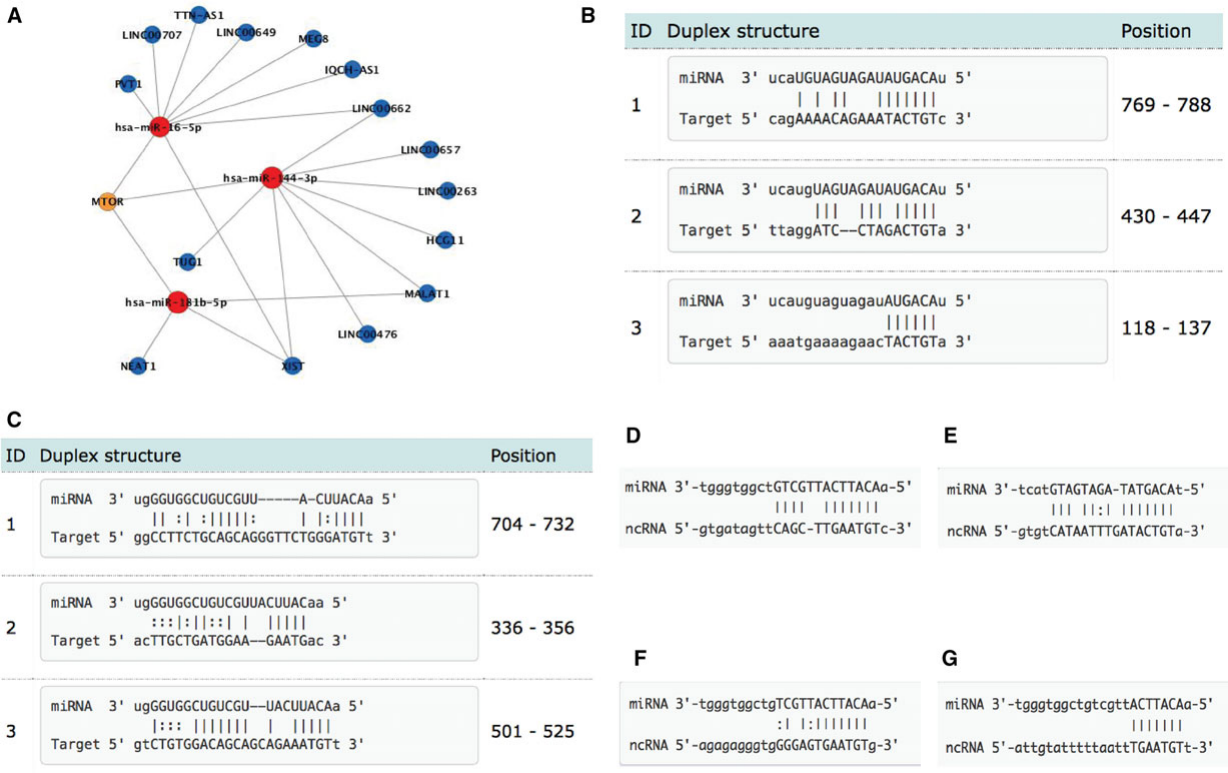
(**A**) mTOR subnetwork. (**B**) Predicted interaction of mir‐144 and mTOR (by miRanda). (**C**) Predicted interaction of mir‐181b‐5p and mTOR (by miRanda). (**D**) Interactions between mir‐181b and MALAT1. (**E**) Interactions between mir‐144 and MALAT1. (**F**) The first interaction site between mir‐181b and NEAT1. (**G**) The second interaction site between mir‐181b and NEAT1. All data were downloaded from Starbase (v2.0) and miRTarBase (v4.5).

In global diabetes‐related ce‐network, we focused on mTOR subnetwork due to the central role of mTOR in energy metabolism. The components of mTOR subnetwork were shown in Figure 4A. miR‐144 and miR‐181b and their predicted or validated binding sites with mTOR were shown in Figure 4B and C 14, 15. We detected the interactions between miR‐181b‐MALAT1 and miR‐144‐MALAT1 (Fig. 4D and E). The two interactions sites between miR‐181b and NEAT1 were shown in Figure 4F and G.

### The ncRNAs‐involved mTOR signalling pathway

**Figure 5:**
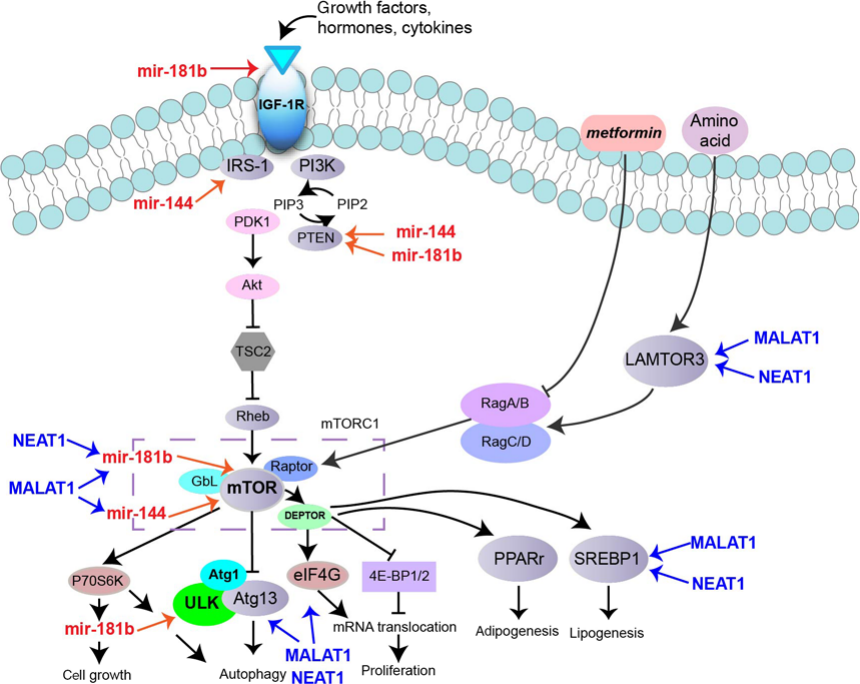
The ncRNAs involved in mTOR signalling pathway. MicroRNAs were labelled in red, and lncRNAs were labelled in blue. MALAT1 and NEAT1 were mapped to mTOR signalling pathway according to a study using proteomics with CHART‐enriched material (CHARTMS) to identify proteins associated with NEAT1 and MALAT1 16. SREBP1, ATG13, LAMTOR3, EIF4G were associated with both MALAT1 and NEAT1.

The mTOR subnetwork extracted from DMCN consisted of mTOR gene, three microRNAs and 15 lncRNAs (Fig. 4A). We focused on miR‐181b and miR‐144 and lncRNAs MALAT1 and NEAT1 due to their potential relationship with diabetes. To annotate these ncRNAs to mTOR signalling pathway, we mapped MALAT1 and NEAT1 to mTOR signalling pathway according to a study using proteomics with CHART‐enriched material (CHARTMS) to identify proteins associated with NEAT1 and MALAT1 *in vivo* 16. The results showed that SREBP1, ATG13, LAMTOR3, EIF4G were associated with both MALAT1 and NEAT1. LAMTOR3 is a member of trimeric protein complex called Ragulator, which is a Rag‐interacting complex essential for amino acid signalling to mTORC1 17 (Fig. 5). In addition, Ragulator‐Rag complex serves as a docking site for mTORC1 on lysosomal membrane, and lysosomes play roles in autophagy, energy metabolism and intracellular signalling 18. Downstream of mTORC1, SREBPs are activated through S6K1 to induce the expression of genes involved in fatty acids and sterols metabolism 19. Another mTOR signalling downstream effector, ATG13, functions as a positive regulator of ULK1 (an autophagy initiating kinase) to suppress initiation of autophagy during nutrient abundance 18. EIF4G acts as a scaffolding protein for translation initiation complex, and insulin signalling regulates translation initiation by regulating eIF4G1 20.

### NEAT1 and miR‐181b levels in HL7720 cells

**Figure 6:**
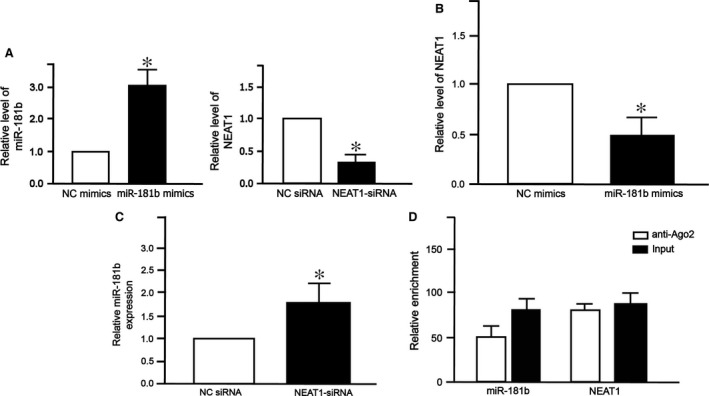
NEAT1 and miR‐181b levels in HL7702 cells. (**A**) Relative miR‐181b and NEAT1 levels in HL7702 cells transfected with negative control (NC) mimics *versus* miR‐181b mimics, or negative control (NC) siRNA *versus* NEAT1 siRNA. (**B**) The expression of NEAT1 in HL7702 cells transfected with miR‐181b mimics. (**C**) The expression of miR‐181b in HL7702 cells transfected with NEAT1‐siRNA. For comparison, expression level in cells transfected with NC mimics or NC siRNA was set to 1. All experiments were repeated three times. Each bar represents the mean ± S.D. of three independent experiments. *P < 0.05. (**D**) RIP assay showing the relative enrichment of AGO2‐immunoprecipitated NEAT1 and mir‐181b. anti‐Ago2: samples incubated with Ago antibody; Input: cell lysate.

After transfection of miR181b mimics into HL7720 cells, we found that miR‐181b level increased (Fig. 6A left) while NEAT1 level decreased (Fig. 6B). On the contrary, after transfection of NEAT‐siRNA into HL7720 cells, NEAT1 level decreased (Fig. 6A right) while the level of miR‐181b was up‐regulated compared to cells transfected with NC siRNA (Fig. 6C). RIP assay showed that the relative enrichment of AGO2‐immunoprecipitated NEAT1 and miR‐181b decreased (Fig. 6D). These results suggest that NAET1 may be one target of miR‐181b.

### The interaction between NEAT1 and miR‐181b by mTOR signalling pathway

**Figure 7:**
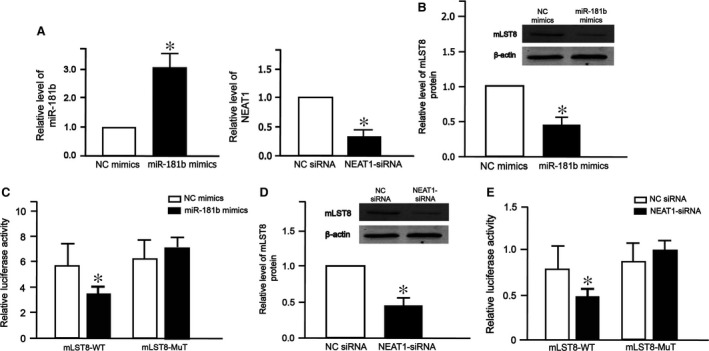
The interaction between NEAT1 and miR‐181b. (**A**) Relative miR‐181b and NEAT1 levels in cells transfected with negative control (NC) mimics *versus* miR‐181b mimics, or negative control (NC) siRNA *versus* NEAT1 siRNA. The expression of mLST8 protein in cells transfected with NC mimics *versus* miR‐181b mimics (**B**) or NC siRNA *versus* NEAT1 siRNA (**D**). The luciferase activity of mLST8 promoter in cells transfected with NC mimics *versus* miR‐181b mimics (**C**) or NC siRNA *versus* NEAT1‐siRNA (**E**). All experiments were repeated three times. Each bar represents the mean ± S.D. of three to six independent experiments. *P < 0.05.

To investigate whether mTOR signalling pathway plays an important role in ceRNA of NEAT1 and miR‐181b, we searched the TargetScan database 7.1 and found that mLST8, a subunit of both mTOR complex 1 (mTORC1) and complex 2 (mTORC2), was a potential target of miR‐181b. As expected, miR‐181b level increased after transfection with miR‐181b mimics (Fig. 7A left), while NEAT1 level decreased after transfection with NEAT‐siRNA (Fig. 7A right). Western blot analysis showed that the expression of mLST8 decreased after transfection with miR‐181b mimics (Fig. 7B). Luciferase assay showed that miR‐181b mimics reduced mLST8‐WT luciferase activity (Fig. 7C). Furthermore, NEAT1‐siRNA decreased protein level of mLST8 (Fig. 7D). Similarly, NEAT1‐siRNA reduced mLST8‐WT luciferase activity (Fig. 7E). These results demonstrate that NEAT1‐miR‐181b‐mLST8 ceRNA pair regulates mTOR signalling pathway.

## Discussion

Accumulating evidence has demonstrated regulatory role of ncRNAs in metabolic disease, and their potential as novel targets for drug development 21, 22. In this study, we constructed a ceRNA network to identify regulatory role of ncRNAs in diabetes.

DMCN contained 98 genes, 167 lncRNAs and 86 microRNAs. As shown in Table 1, the most connected genes were VEGFA and ESR1, consistent with that allelic variants of VEGFA gene are associated with microvascular complications of diabetes and atherosclerosis 23 and that ESR1 gene was one of the most highly interconnected T2DM network ‘hub’ genes 24. LncRNA MIR22HG was at top of the degree list. A study reported that MIR22HG was significantly up‐regulated in the endothelial cell in response to hypoxia both *in vitro* and *in vivo* 25. Another study reported that MIR22HG responded to chemical stresses in induced pluripotent stem cells, indicating its role in response to external stimulus 26. OIP5‐AS1 is highly expressed in the nervous system and is important for neurogenesis during development 27. However, the relationship between other lncRNAs and diabetes mellitus has not been reported.

Degree and betweenness are two major parameters to characterize DMCN. Among the top 10 microRNAs with the highest degree, hsa‐mir‐21 was at top of the list (degree = 35). miR‐21 was reported to be positively correlated with BMI 28. Moreover, miR‐21 has been shown to contribute to cytokine‐mediated β‐cell dysfunction in type 1 diabetes 29. miR‐155 (degree = 23) played a role in linking adipose tissue dysfunction and the development of obesity‐associated disorders including type 2 diabetes 30. We also calculated a network feature‐node betweenness, defined as the fraction of shortest paths between node pairs that pass through a given node 31. The betweenness of a microRNA represents its role in other nodes that connect to each other. Our results showed that miR‐21, miR‐155, miR‐30a had not only higher degrees, but also higher betweenness values (Table 1), confirming the strong correlation between degree and betweenness. However, miR‐632 and miR‐99a had the highest betweenness value (betweenness value = 1) but relative low degree, suggesting their role in connecting genes and lncRNAs rather than being a ‘hub’ node in a local network.

To investigate the functions of T2DM‐related genes, we annotated these genes into GO and KEGG analysis. We found that the most significant terms were involved in the regulation of cell death. Apoptosis of β‐cells has been demonstrated to be involved in autoimmune T1DM and T2DM 32. Moreover, apoptosis can lead to pancreatic β‐cell loss and endothelial cell injury 33, suggesting its significant role in the pathogenesis in DM and its complications.

Due to its central role in energy metabolism, we extracted mTOR subnetwork from the global DMCN and aimed to investigate the regulatory role of ncRNAs in mTOR pathway. As shown in Figure 5A, 15 lncRNAs including MALAT1, NEAT1 and three microRNAs hsa‐mir‐144‐3p, hsa‐mir‐181b‐5p and hsa‐mir‐16‐5p were grouped in this subnetwork. We only focused on lncRNA MALAT1 and NEAT1 considering their potential role in diabetes. mTOR is an atypical serine/threonine protein kinase that integrates various extracellular signals into a variety of anabolic processes, cell growth and autophagy 34. Dysregulation in mTOR signalling is implicated in various diseases such as obesity, T2DM, cancer and ageing 35.

Recently, various studies showed that microRNAs affect mTOR pathway by targeting upstream regulators including IGF‐R, PI3K and Akt 36. In our study, we found that miR‐144 and miR‐181b were involved in the regulation of mTOR signalling by targeting both upstream and downstream effectors. *In silico* analysis has revealed two miR‐144 binding sites in the mTOR 3′ untranslated region, and *in vitro* assays showed that mTOR is a direct target of mir‐144 37. Increased circulating level of miR‐144 has been found to inhibit insulin receptor substrate 1(IRS‐1) to impair insulin signalling in T2DM 38. Insulin growth factors (IGF‐1 and IGF‐2) and their receptors play a significant role in insulin resistance and T2DM, and miR‐181b overexpression inhibited PI3K/AKT signalling through targeting IGF‐1R 21. lncRNA metastasis‐associated lung adenocarcinoma transcript 1 (MALAT1) was associated with diabetes‐induced microvascular dysfunction, as up‐regulation of MALAT1 could activate p38/MAPK signalling and regulate retinal endothelial cell function under diabetic condition 39. A recent study indicated that MALAT1 regulated hyperglycaemia induced inflammatory process in endothelial cells 40. NEAT1 (nuclear enriched abundant transcript 1) is a nuclear lncRNA that was shown to participate in miR‐140 induced adipogenesis 41.

Then, we annotated these ncRNAs to mTOR signalling pathway. We further validated two ceRNA pairs (MALAT1‐miR‐144‐mTOR and NEAT1‐miR‐181b‐mTOR) involved in mTOR pathway and explored their significance in diabetes mellitus. NEAT1 is positively co‐expressed with mLST8, and mLST8 expression was decreased by mir‐181b mimics, indicating potential communication between NEAT1 and mTOR signaling pathway medicated by microRNA‐target sites. These findings establish a novel connection between lncRNA and microRNA in diabetes mellitus.

In summary, we constructed a T2DM‐related ceRNA network (DMCN) to explore the biological functions of lncRNAs during the development of diabetes mellitus. A mTOR‐centred ceRNA subnetwork was extracted, and we experimentally validated that NEAT1 was potentially communicated with mTOR signalling pathway target protein mLST8 *via* the association with miR‐181b. These findings provide significant insight into lncRNA regulatory network in T2DM.

## Conflict of interest

None.

## Supporting information


**Figure S1** Schematic figure of construction of T2DM related ceRNA network (DMCN).
**Figure S2** Example network with five nodes and five edges.

## References

[1] Unnikrishnan R , Anjana RM , Mohan V . Diabetes mellitus and its complications in India. Nat Rev Endocrinol. 2016; 12: 357–70. 27080137 10.1038/nrendo.2016.53

[2] Price NL , Ramirez CM , Fernández‐Hernando C . Relevance of microRNA in metabolic diseases. Crit Rev Clin Lab Sci. 2014; 51: 305–20. 25034902 10.3109/10408363.2014.937522

[3] Vienberg S , Geiger J , Madsen S , *et al* MicroRNAs in Metabolism. Acta Physiol. 2017; 219: 346–361. 10.1111/apha.12681 PMC5297868 27009502

[4] Salmena L , Poliseno L , Tay Y , *et al* A ceRNA hypothesis: the Rosetta Stone of a hidden RNA language? Cell. 2011; 146: 353–8. 21802130 10.1016/j.cell.2011.07.014 PMC3235919

[5] Li JH , Liu S , Zhou H , *et al* starBase v2.0: decoding miRNA‐ceRNA, miRNA‐ncRNA and protein‐RNA interaction networks from large‐scale CLIP‐Seq data. Nucleic Acids Res. 2014; 42: D92–7. 24297251 10.1093/nar/gkt1248 PMC3964941

[6] Sumazin P , Yang X , Chiu HS , *et al* *et al* An extensive microRNA‐mediated network of RNA‐RNA interactions regulates established oncogenic pathways in glioblastoma. Cell. 2011; 147: 370–81. 22000015 10.1016/j.cell.2011.09.041 PMC3214599

[7] Cesana M , Cacchiarelli D , Legnini I , *et al* A long noncoding RNA controls muscle differentiation by functioning as a competing endogenous RNA. Cell. 2011; 147: 358–69. 22000014 10.1016/j.cell.2011.09.028 PMC3234495

[8] Costa V , Esposito R , Aprile M , *et al* Non‐coding RNA and pseudogenes in neurodegenerative diseases: “The (un)Usual Suspects”. Front Genet. 2012; 3: 231. 23118739 10.3389/fgene.2012.00231 PMC3484327

[9] Dai HJ , Wu JC , Tsai RT , *et al* T‐HOD: a literature‐based candidate gene database for hypertension, obesity and diabetes. Database (Oxford). 2013; 2013: bas061. 23406793 10.1093/database/bas061 PMC3570736

[10] Hamosh A , Scott AF , Amberger JS , *et al* Online Mendelian Inheritance in Man (OMIM), a knowledgebase of human genes and genetic disorders. Nucleic Acids Res. 2005; 33: D514–7. 15608251 10.1093/nar/gki033 PMC539987

[11] Vergoulis T , Vlachos IS , Alexiou P , *et al* TarBase 6.0: capturing the exponential growth of miRNA targets with experimental support. Nucleic Acids Res. 2012; 40: D222–9. 22135297 10.1093/nar/gkr1161 PMC3245116

[12] Hsu SD , Tseng YT , Shrestha S , *et al* miRTarBase update 2014: an information resource for experimentally validated miRNA‐target interactions. Nucleic Acids Res. 2014; 42: D78–85. 24304892 10.1093/nar/gkt1266 PMC3965058

[13] da Huang W , Sherman BT , Lempicki RA . Systematic and integrative analysis of large gene lists using DAVID bioinformatics resources. Nat Protoc. 2009; 4: 44–57. 19131956 10.1038/nprot.2008.211

[14] Chou CH , Chang NW , Shrestha S , *et al* miRTarBase 2016: updates to the experimentally validated miRNA‐target interactions database. Nucleic Acids Res. 2016; 44: D239–47. 26590260 10.1093/nar/gkv1258 PMC4702890

[15] Helwak A , Kudla G , Dudnakova T , *et al* Mapping the human miRNA interactome by CLASH reveals frequent noncanonical binding. Cell. 2013; 153: 654–65. 23622248 10.1016/j.cell.2013.03.043 PMC3650559

[16] West JA , Davis CP , Sunwoo H , *et al* The long noncoding RNAs NEAT1 and MALAT1 bind active chromatin sites. Mol Cell. 2014; 55: 791–802. 25155612 10.1016/j.molcel.2014.07.012 PMC4428586

[17] Sancak Y , Bar‐Peled L , Zoncu R , *et al* Ragulator‐Rag complex targets mTORC1 to the lysosomal surface and is necessary for its activation by amino acids. Cell. 2010; 141: 290–303. 20381137 10.1016/j.cell.2010.02.024 PMC3024592

[18] Huang K , Fingar DC . Growing knowledge of the mTOR signaling network. Semin Cell Dev Biol. 2014; 36: 79–90. 25242279 10.1016/j.semcdb.2014.09.011 PMC4253687

[19] Duvel K , Yecies JL , Menon S , *et al* Activation of a metabolic gene regulatory network downstream of mTOR complex 1. Mol Cell. 2010; 39: 171–83. 20670887 10.1016/j.molcel.2010.06.022 PMC2946786

[20] Liew CW , Assmann A , Templin AT , *et al* Insulin regulates carboxypeptidase E by modulating translation initiation scaffolding protein eIF4G1 in pancreatic β cells. Proc Natl Acad Sci USA. 2014; 111: E2319–28. 24843127 10.1073/pnas.1323066111 PMC4050564

[21] Chakraborty C , Doss CG , Bandyopadhyay S , *et al* Influence of miRNA in insulin signaling pathway and insulin resistance: micro‐molecules with a major role in type‐2 diabetes. Wiley Interdiscip Rev RNA. 2014; 5: 697–712. 24944010 10.1002/wrna.1240

[22] Latronico MV , Condorelli G . Therapeutic applications of noncoding RNAs. Curr Opin Cardiol. 2015; 30: 213–21. 25768956 10.1097/HCO.0000000000000162

[23] Qu L , He B , Pan Y , *et al* Association between polymorphisms in RAPGEF1, TP53, NRF1 and type 2 diabetes in Chinese Han population. Diabetes Res Clin Pract. 2011; 91: 171–6. 21146886 10.1016/j.diabres.2010.11.019

[24] Hale PJ , López‐Yunez AM , Chen JY . Genome‐wide meta‐analysis of genetic susceptible genes for Type 2 Diabetes. BMC Syst Biol. 2012; 6 Suppl 3: S16. 10.1186/1752-0509-6-S3-S16 PMC3524015 23281828

[25] Voellenkle C , Garcia‐Manteiga JM , Pedrotti S , *et al* Implication of Long noncoding RNAs in the endothelial cell response to hypoxia revealed by RNA‐sequencing. Sci Rep. 2016; 6: 24141. 27063004 10.1038/srep24141 PMC4827084

[26] Tani H , Onuma Y , Ito Y , *et al* Long non‐coding RNAs as surrogate indicators for chemical stress responses in human‐induced pluripotent stem cells. PLoS One. 2014; 9: e106282. 25171338 10.1371/journal.pone.0106282 PMC4149554

[27] Ulitsky I , Shkumatava A , Jan CH , *et al* Conserved function of lincRNAs in vertebrate embryonic development despite rapid sequence evolution. Cell. 2011; 147: 1537–50. 22196729 10.1016/j.cell.2011.11.055 PMC3376356

[28] Ling H , Li X , Yao CH , *et al* The physiological and pathophysiological roles of adipocyte miRNAs. Biochem Cell Biol. 2013; 91: 195–202. 23859012 10.1139/bcb-2012-0053

[29] Dumortier O , Hinault C , Van Obberghen E . MicroRNAs and metabolism crosstalk in energy homeostasis. Cell Metab. 2013; 18: 312–24. 23850315 10.1016/j.cmet.2013.06.004

[30] Kloting N , Berthold S , Kovacs P , *et al* MicroRNA expression in human omental and subcutaneous adipose tissue. PLoS One. 2009; 4: e4699. 19259271 10.1371/journal.pone.0004699 PMC2649537

[31] Diez D , Wheelock AM , Goto S , *et al* The use of network analyses for elucidating mechanisms in cardiovascular disease. Mol BioSyst. 2010; 6: 289–304. 20094647 10.1039/b912078e

[32] Vives‐Pi M , Rodriguez‐Fernandez S , Pujol‐Autonell I . How apoptotic beta‐cells direct immune response to tolerance or to autoimmune diabetes: a review. Apoptosis. 2015; 20: 263–72. 25604067 10.1007/s10495-015-1090-8 PMC4328127

[33] Maiese K . Programming apoptosis and autophagy with novel approaches for diabetes mellitus. Curr Neurovasc Res. 2015; 12: 173–88. 25742566 10.2174/1567202612666150305110929 PMC4380829

[34] Laplante M , Sabatini DM . mTOR signaling in growth control and disease. Cell. 2012; 149: 274–93. 22500797 10.1016/j.cell.2012.03.017 PMC3331679

[35] Cornu M , Albert V , Hall MN . mTOR in aging, metabolism, and cancer. Curr Opin Genet Dev. 2013; 23: 53–62. 23317514 10.1016/j.gde.2012.12.005

[36] Santulli G , Totary‐Jain H . Tailoring mTOR‐based therapy: molecular evidence and clinical challenges. Pharmacogenomics. 2013; 14: 1517–26. 24024901 10.2217/pgs.13.143 PMC4186793

[37] Iwaya T , Yokobori T , Nishida N , *et al* Downregulation of miR‐144 is associated with colorectal cancer progression *via* activation of mTOR signaling pathway. Carcinogenesis. 2012; 33: 2391–7. 22983984 10.1093/carcin/bgs288

[38] Karolina DS , Armugam A , Tavintharan S , *et al* MicroRNA 144 impairs insulin signaling by inhibiting the expression of insulin receptor substrate 1 in type 2 diabetes mellitus. PLoS One. 2011; 6: e22839. 21829658 10.1371/journal.pone.0022839 PMC3148231

[39] Liu JY , Yao Yao , Li XM . *et al* Pathogenic role of lncRNA‐MALAT1 in endothelial cell dysfunction in diabetes mellitus. Cell Death Dis. 2014; 5: e1506. 25356875 10.1038/cddis.2014.466 PMC4649539

[40] Puthanveetil P , Chen S , Feng B , *et al* Long non‐coding RNA MALAT1 regulates hyperglycaemia induced inflammatory process in the endothelial cells. J Cell Mol Med. 2015; 19: 1418–25. 25787249 10.1111/jcmm.12576 PMC4459855

[41] Wei S , Du M , Jiang Z , *et al* Long noncoding RNAs in regulating adipogenesis: new RNAs shed lights on obesity. Cell Mol Life Sci. 2016; 73: 2079–87. 26943803 10.1007/s00018-016-2169-2 PMC5737903

