# Supplementary material for: Construction of competitive endogenous RNA network reveals regulatory role of long non‐coding RNAs in type 2 diabetes mellitus
Source: J Cell Mol Med. 2017 Jun 23;21(12):3204–13. doi: 10.1111/jcmm.13224 (PMC5706502; doi:10.1111/jcmm.13224)
Supplement: Supplementary file 1 — Figure S1 Schematic figure of construction of T2DM related ceRNA network (DMCN). Figure S2 Example network with five nodes and five edges. [file JCMM-21-3204-s001.doc]

**Supplementary files**


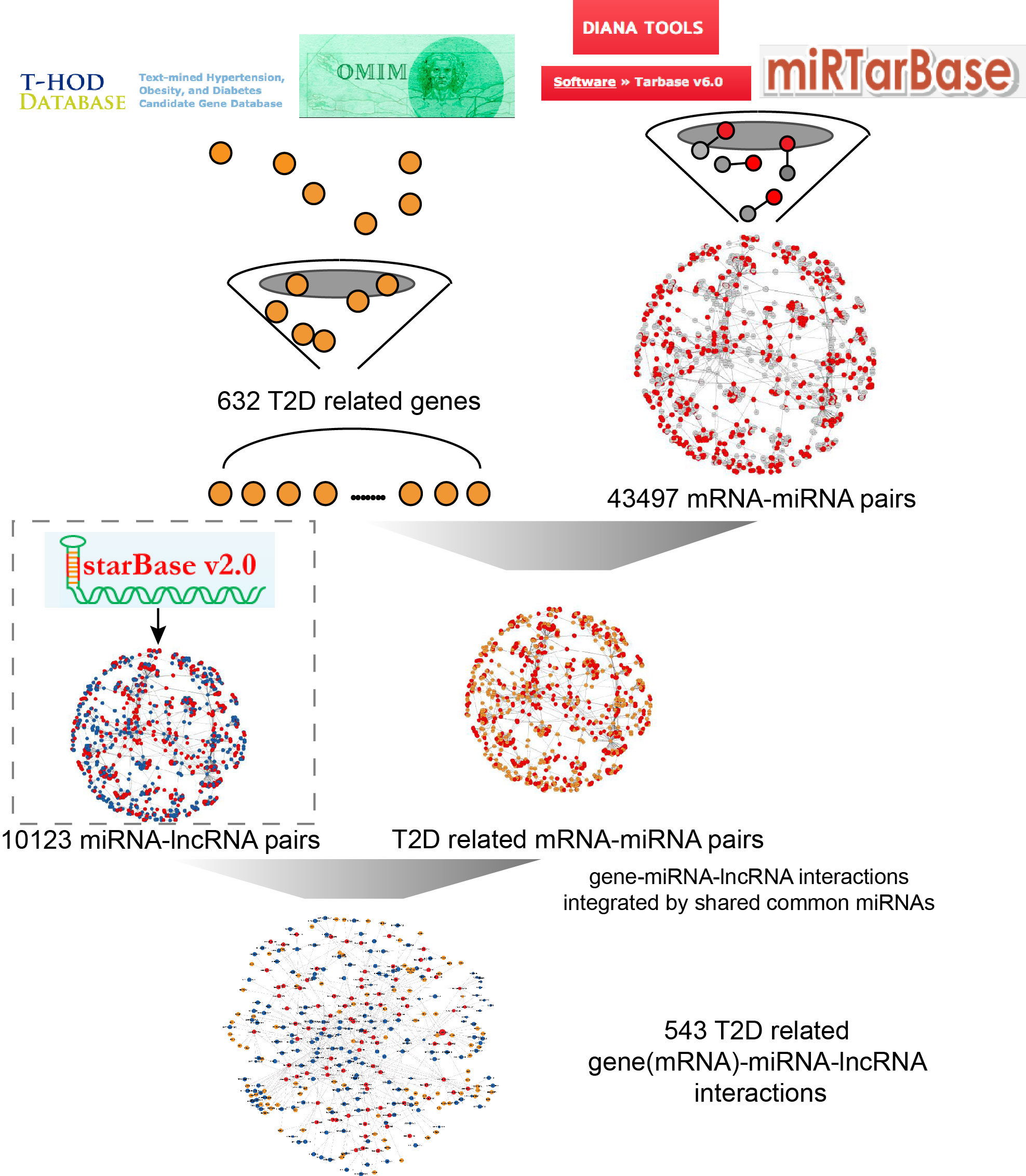


Figure S1. Schematic figure of construction of T2DM related ceRNA network (DMCN). First, we obtained 632 human T2D-related genes (labeled as yellow nodes) from T-HOD and OMIM’s Morbid Map. Second, we identified microRNA and T2D related gene pairs (labeled as red-grey nodes pairs) and integrated these T2D-related genes into these microRNA-mRNA pairs. Then we obtained a T2D related mRNA-miRNA pairs network (labeled with yellow and red nodes network). Next, human microRNA-lncRNA interaction pairs were identified from starBase (V2.0) (labeled with red and blue nodes network), and we integrated two networks through shared common microRNAs and constructed DMCN (543 T2D related gene-miRNA-lncRNA interactions labled with yellow-red-blue nodes network).


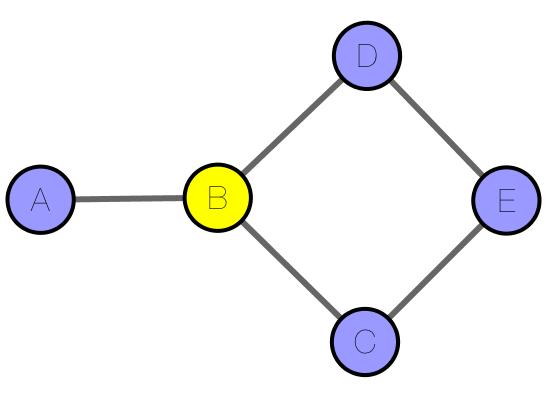


Figure S2. Example network with five nodes and five edges.

In undirected networks, the node degree of a node *i* is the number of edges linked to *i*. For example, the degree of node A is 1, while the degree of B is 3, and the degree of C, D and E is 2. The betweenness of a node reflects the amount of control that this node exerts over the interactions of other nodes in the network [1]. This measure favors nodes that join communities (dense subnetworks), rather than nodes that lie inside a community. Nodes with higher betweenness indicate that they are prone to regulate key pathways. The betweenness Bi stands for a node I is computed as follows:

$$B_{i}=\frac{1}{\left(n-1\right)\left(n-2\right)}\sum_{s\neq i\neq t} \frac{s_{st}\left(i\right)}{s_{st}}$$

where S_st_ is the number of shortest paths from s to t, and S_st_ (i) represents the number of shortest paths from s to t that pass through a node i. The measure is normalized by the number of pairs of nodes except i, which is (n-1)(n-2)，where n is the total number of nodes in the connected component that n belongs to. Thus, the betweenness of each node is a number between 0 and 1. For example, the betweenness of node B is computed as follows:$Bi=\frac{7}{(5-1)(5-2)}\approx0.58$

**Reference** 1. Yoon, J., A. Blumer, and K. Lee*, An algorithm for modularity analysis of directed and weighted biological networks based on edge-betweenness centralit*y. Bioinformatics, 2006**.** 22(24): 3106-8.
